# Renal cryoablation combined with prior transcatheter arterial embolization in non-dialysis patients with stage 4 or 5 chronic kidney disease: a retrospective study

**DOI:** 10.1007/s11604-023-01416-z

**Published:** 2023-04-01

**Authors:** Noriyuki Umakoshi, Toshihiro Iguchi, Yusuke Matsui, Koji Tomita, Mayu Uka, Takahiro Kawabata, Kazuaki Munetomo, Shoma Nagata, Hideo Gobara, Motoo Araki, Takao Hiraki

**Affiliations:** 1 Department of Radiology, Okayama University Hospital, 2-5-1 Shikata-Cho, Kitaku, Okayama 700-8558 Japan; 2 Deptartment of Radiological Technology, Okayama University Graduate School of Health Science, Okayama, Japan; 3 Department of Radiology, Faculty of Medicine, Dentistry and Pharmaceutical Sciences, Okayama University, 2-5-1 Shikata-Cho Kita-Ku, Okayama, 700-8558 Japan; 4 Division of Medical Informatics, Okayama University Hospital, 2-5-1 Shikata-Cho Kita-Ku, Okayama, 700-8558 Japan; 5 Department of Urology, Faculty of Medicine, Dentistry and Pharmaceutical Sciences, Okayama University, 2-5-1 Shikata-Cho Kita-Ku, Okayama, 700-8558 Japan

**Keywords:** Renal cryoablation, Transcatheter arterial embolization, Chronic kidney disease

## Abstract

**Purpose:**

To retrospectively evaluate cryoablation combined with prior transcatheter arterial embolization (TAE) for renal cell carcinoma (RCC) in non-dialysis patients with stage 4 or 5 chronic kidney disease (CKD).

**Materials and methods:**

Patients with stage 4 or 5 CKD undergoing TAE and cryoablation for RCC between May 2012 and October 2021 were included. TAE was selectively performed using iodized oil with absolute ethanol or gelatin sponge 1–14 days before cryoablation. Local efficacy, safety, and changes in renal function were evaluated.

**Results:**

Nine patients (seven men and two women; median age, 64 years; range 52–88 years) with nine RCCs (mean diameter, 3.0 ± 1.0 cm; range 1.7–4.7 cm) were included. The mean pre-treatment estimated glomerular filtration rate (eGFR) was 24.2 ± 5.6 ml/min/1.73 m^2^ (range 10.4–29.2 ml/min/1.73 m^2^). The mean amount of contrast medium used in TAE was 58 ± 29 ml (range 40–128 ml). Except in one patient (grade 3 pyelonephritis), no grade ≥ 3 complications occurred. During the follow-up period (median, 18 months; range 7–54 months), no local tumor progression occurred. In two patients with pre-treatment eGFR of < 20 ml/min/1.73 m^2^, hemodialysis was initiated at 3 and 19 months after cryoablation. At their last follow-up, the remaining seven patients showed a decrease of 6.2 ± 5.3 ml/min/1.73 m^2^ (range 0.7–17.2 ml/min/1.73 m^2^) in their eGFR.

**Conclusion:**

Cryoablation combined with TAE for RCC in non-dialysis patients with stage 4 or 5 CKD was effective and safe, with an acceptable impact on renal function.

## Introduction

Thermal ablation therapy (e.g., radiofrequency, microwave, and cryoablation) has been recently used to treat small renal cell carcinoma (RCC), mainly in inoperable patients. Radiofrequency and cryoablation are the most studied ablative technologies [1]. Cryoablation is a safe and effective therapy because the ablation zone is confirmed as an ice ball, and the rates of 5-year overall survival, cause-specific survival, and progression-free survival are reported to be 84.8%–97.8%, 94.3%–100%, and 85.9%–100%, respectively [2–5].

The most typical type of RCC (i.e., clear cell RCC) is usually hypervascular, which may benefit from transarterial embolization (TAE) before thermal ablation therapy, similar to hepatocellular carcinoma, to improve oncologic outcomes [6, 7]. In addition, it is theorized that TAE for RCC before ablation can reduce the risk of seeding and bleeding [8]. Furthermore, when using iodized oil for TAE, tumor localization on computed tomography (CT) during percutaneous ablation may be enhanced [6, 8]. Some studies have already shown that the combination of TAE and percutaneous ablation is technically feasible, safe, and oncologically effective [9–12].

Although cryoablation of RCC may be a candidate treatment for patients with various comorbidities such as chronic kidney disease (CKD), the use of iodized contrast medium for severe CKD patients is generally contraindicated because of the risk of contrast-induced nephropathy [13]. Additionally, TAE may raise concerns about the deterioration of renal function in CKD patients. Some authors have reported the outcomes of renal cryoablation in patients with CKD at various stages [14–16]. However, no studies have reported cryoablation combined with prior TAE in RCC patients with severe (i.e., stage 4 or 5) CKD preparing for renal replacement therapy. We hypothesized that this combination therapy would be effective and safe even for patients with high-stage CKD, and retrospectively evaluated its outcomes, including the effect on renal function.

Therefore, this study aimed to retrospectively evaluate the efficacy, safety, and changes in renal function of cryoablation combined with prior TAE for RCC in non-dialysis patients with stage 4 or 5 CKD.

## Materials and methods

This retrospective study [approval number, KEN2202-007] was approved by our ethics committee, and opt-out consent was obtained for the retrospective use of patient data. This study was conducted in accordance with the Declaration of Helsinki. We obtained written informed consent for cryoablation and TAE from all patients before treatment.

### Inclusion and exclusion criteria

The following patients were included: (1) those undergoing cryoablation combined with TAE within two weeks before cryoablation between May 2012 and October 2021 at our institution, and (2) those with stage 4 or 5 CKD (i.e., those with estimated glomerular filtration rate [eGFR] < 30 ml/min/1.73 m^2^ within one month before TAE). Patients on dialysis were excluded.

### Renal cryoablation combined with prior transcatheter arterial embolization

The following indications for cryoablation combined with TAE were determined in consensus by experienced interventional radiologists at a pre-treatment conference: parenchymal type RCCs and > 3-cm lesions were selected for TAE. As a preventive measure for contrast-induced nephropathy, we performed a fluid challenge from the day before TAE until several hours after the procedure without contraindications to volume expansion, depending on the patient’s renal function.

**Fig. 1:**
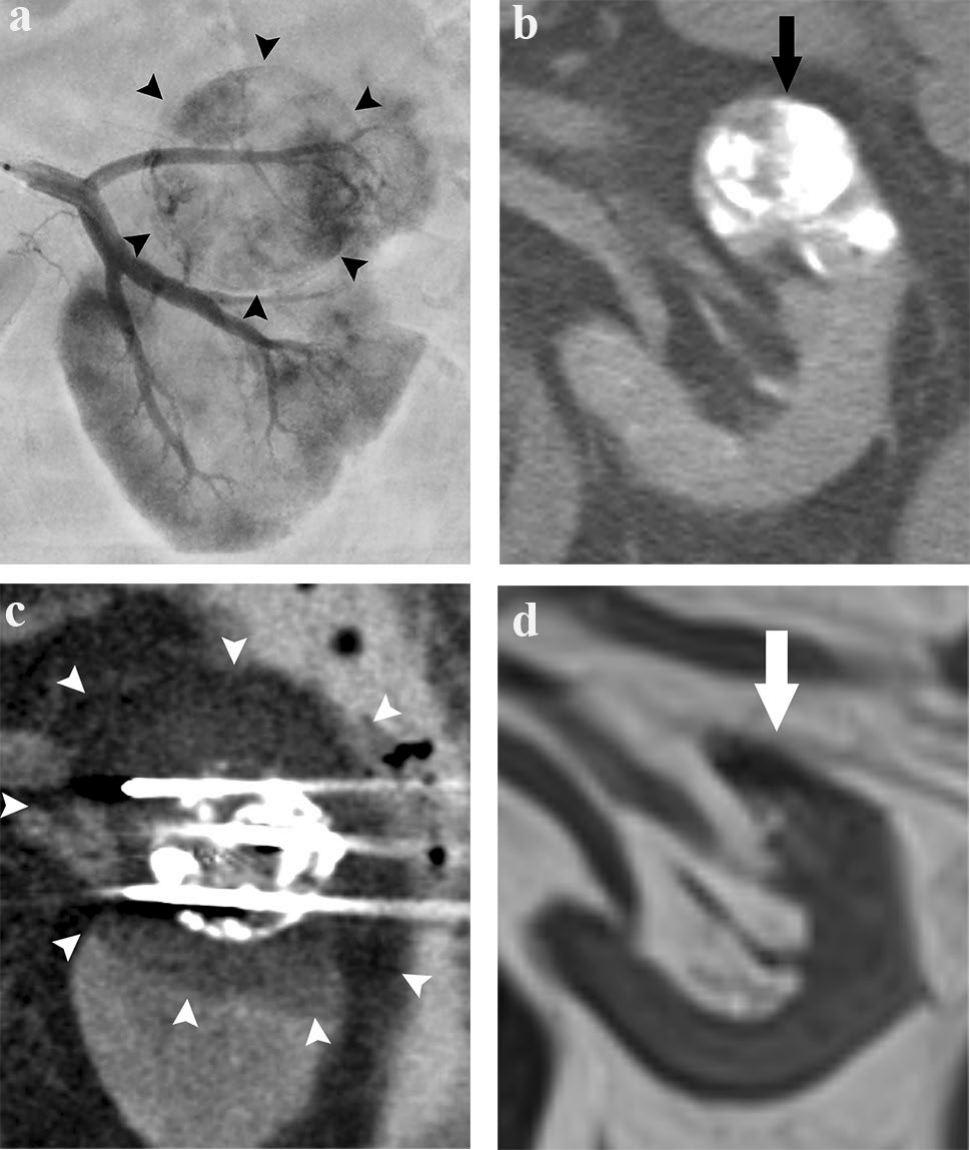
A 73-year-old man with chronic kidney disease, stage 4 (pre-treatment eGFR: 28.4 ml/ min/1.73 m^2^). **a** Angiogram in the left renal artery shows tumor staining (black arrowheads). **b** Unenhanced axial CT images immediately after embolization shows a target renal mass (black arrow) of 2.7 cm in diameter with a focal high-density area due to lipiodol accumulation. **c** Unenhanced coronal CT image immediately after cryoablation shows cryoprobes and an ice ball (arrowheads) covering the tumor circumferentially. **d** Unenhanced coronal T2-weighted MRI image obtained 4.5 years after cryoablation shows shrinkage of the ablation zone (white arrow) without local tumor progression. His eGFR value was 19.1 ml/ min/1.73 m^2^

TAE using iohexol or iopromide was performed by seven interventional radiologists with 4–20 years of experience. A 4-F catheter was inserted via the common femoral artery through a 4-F sheath introducer. After selecting the renal artery, digital subtraction angiography was performed to evaluate and detect the supplying vessels of the target RCC (Fig. 1a). The tumor-feeding arteries were selected using a 1.5- to 2.7-F microcatheter. Embolization was then performed under fluoroscopy to prevent the reflux of embolic agents into non-target vessels. The embolic agents used were a 7:3 mixture of absolute ethanol and iodized oil [6] or a combination of iodized oil and gelatin sponge. The embolic material was injected until stasis of the blood flow in the feeding artery was achieved (Fig. 1b). The extent of embolization was maintained within the therapeutic area assumed in cryoablation as much as possible. Coil embolization was additionally performed if there was a sizable arterial branch across the planned puncture route of the cryoprobe.

Five interventional radiologists with 12–26 years of experience performed cryoablation under local anesthesia using an argon-based cryoablation system (CryoHit®, Galil Medical, Youknum, Israel or VISUAL ICE™, Boston Scientific, Marlborough, MA) with cryoprobes (IceRod™, IceSeed™ or IceRod 1.5 Plus™, Galil Medical). The number and type of cryoprobes used were determined depending on tumor size and location by experienced interventional radiologists based on their consensus. The array of cryoprobes inserted in the tumors was designed to avoid damage to the normal renal parenchyma.

Before cryoablation, a ureteral stent was placed to avoid ureteral freezing injury when the RCC was close to the ureter. When the tumor was adjacent to the organs at risk around the kidney (e.g., the colon, duodenum, and pancreas), water with or without contrast media was injected to keep non-target organs out of the expected ablation zone (i.e., hydro-dissection). After insertion of the cryoprobes under CT fluoroscopy guidance (Aquilion 64, or Aquilion ONE 320, Canon Medical Systems, Otawara, Japan), cryoablation was performed in two 10- to 15-min freezing cycles, separated by two or more minutes of passive thawing [17]. After each freezing, the operator ensured that the tumor was contained within a low attenuation area (“ice-ball”) on the CT with as adequate a circumferential ablation margin (≥ 6 mm from the tumor) as possible (Fig. 1c). If the ablation zone was insufficient to cover the entire tumor, additional freeze and thaw cycles were applied after repositioning the cryoprobes.

Technical success of this combined therapy for RCC was defined as both disappearance of tumoral blush on postembolization angiography in the TAE session and complete coverage of the target during the ablation session.

### Data collection and statistical analysis

Tumor characteristics were evaluated on pre-procedural CT or magnetic resonance imaging (MRI) images. CT was performed in all cases, 7 without contrast and 2 with contrast. An additional non-enhanced MRI was also obtained in 6 cases.The tumor location was categorized as exophytic, central, mixed, and parenchymal based on the definition by Gervais et al. [18]. Additionally, the R.E.N.A.L. nephrometry (RENAL) score [19] was calculated and classified as low (4–6 points), intermediate (7–9 points), or high (10–12 points). Change in renal function was evaluated based on eGFR values. eGFR was calculated using the following formula: eGFR (ml/min/1.73 m^2^) = 194 × SCr^−1.094^ × age^−0.287^ (× 0.739, if female) [20]. The change in eGFR, compared with baseline (i.e., value before TAE), was defined as an eGFR decrease. The percentage of eGFR decrease was defined as the eGFR decrease rate. Complications that occurred during and after the procedure and local tumor progression after cryoablation combined with TAE were also evaluated and categorized according to the Common Terminology Criteria for Adverse Events, version 5.0. Local tumor progression was defined as the appearance of a nodular focus within or adjacent to the ablation zone on follow-up CT or MR images [21].

Categorical data are expressed as raw numbers, proportions, and percentages. Quantitative variables are expressed as means, standard deviations (SD), medians, and ranges. Microsoft Excel 2019 (Microsoft) was used for descriptive statistics. The Wilcoxon signed-rank test was used to compare changes in eGFR before and after cryoablation combined with TAE. P-values < 0.05 were considered to indicate statistically significant differences. Statistical analyses were performed using SPSS software (version 26; IBM Corp., Armonk, NY).

## Results

**Fig. 2:**
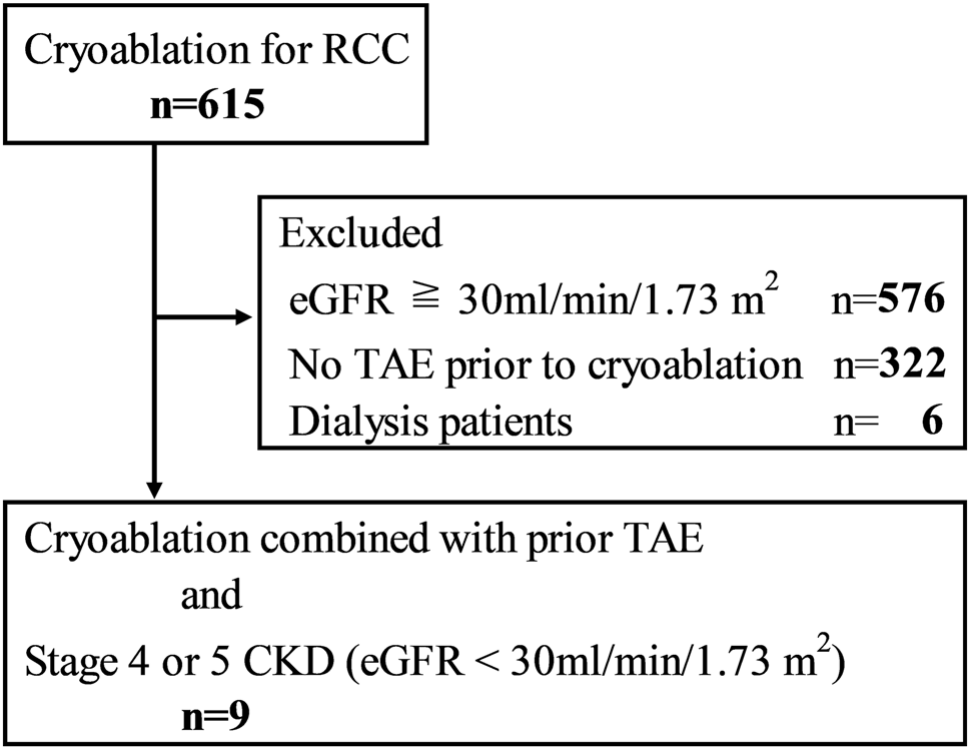
Flow chart depicting patient selection. ※RCC = renal cell carcinoma, eGFR = estimated glomerular filtration rate, TAE = transcatheter arterial embolization, CKD = chronic kidney disease

Between May 2012 and October 2021, percutaneous cryoablation was performed for 615 RCCs. Of these, nine patients (seven men and two women; mean age, 66.4 ± 11.4 years, median 64 years; range 52–88 years) with nine RCCs were included (Fig. 2 and Table 1). The mean eGFR of the patients before TAE was 24.2 ± 5.6 ml/min/1.73 m^2^ (range 10.4–29.2 ml/min/1.73 m^2^).

**Table 1:** Characteristics of nine renal tumors

Variable		Value
Age (years)	Mean ± SD (range)	66.4 ± 11.4 (52–88)
Sex	Man/woman	7/2
Number of kidneys	1/2	3/6
eGFR (mL/min/1.73 m^2^)	Mean ± SD (range)	24.2 ± 5.6 (10.4–29.2)
Tumor diameter (cm)	Mean ± SD (range)	3.0 ± 1.0 (1.7–4.7)
Laterality	Right/left	3/6
Histology	Clear cell RCC/non-biopsy	7/2
RENAL score	Low/intermediate/high	1/7/1

*SD* standard deviation, *eGFR* estimated glomerular filtration rate, *RCC* renal cell carcinoma, *RENAL score* R.E.N.A.L. nephrometry score

Seven tumors were histologically diagnosed as clear cell RCC by percutaneous biopsy. The remaining two tumors did not undergo biopsy and were clinically diagnosed as RCC. The former had developed in a patient with von Hippel-Lindau disease and a resected clear cell RCC and the latter in patients with a resected chromophobe RCC. The mean tumor diameter was 3.0 ± 1.0 cm (median, 2.8 cm; range, 1.7–4.7 cm). The RENAL score was low in one tumor, intermediate in seven, and high in one.

TAE was performed for lesions of the parenchymal type (5/9 tumors, 56%), > 3 cm lesions (3/5 tumors, 33%), and both (1/9 tumors, 11%). All nine patients successfully underwent TAE within 1–14 days (mean 3.8 ± 4.2, median 1 day) before cryoablation (Table 2). A mixture of iodized oil and absolute ethanol (7/9 patients, 78%) or a combination of iodized oil and gelatin sponge (2/9 patients, 22%) was used as an embolic material. Additional coil embolization was performed in 2 (22%) of 9 patients. We used 58 ± 29 ml (range 40–128 ml) of contrast medium during TAE.

**Table 2:** Details of the TAE and cryoablation procedure

Variable		Value
TAE		
Embolic materials	Iodized oil with absolute ethanol	5
	Iodized oil with absolute ethanol + coil	2
	Iodized oil + gelatin sponge	2
Amount of contrast medium (ml)	Mean ± SD (range)	58 ± 29 (40–128)
Days from TAE to cryoablation	Mean ± SD (range)	3.8 ± 4.2 (1–14)
Cryoablation		
Number of cryoprobes used	3/4	8/1
Ureteral stent placement	Yes/no	2/7
Hydro-dissection	Yes/no	7/2
Total freezing time (min)	Mean ± SD (range)	42 ± 16 (30–75)

*SD* standard deviation, *TAE* transcatheter arterial embolization

Eight RCCs used three cryoprobes, and one RCC used four cryoprobes. The mean total freezing time was 42 ± 16 min (range 30–75 min). Ureteral stent placement and hydro-dissection were performed in two and seven cryoablation sessions. Cryoablation was technically successful for all RCCs. One patient developed pyelonephritis (grade 3) 16 days after cryoablation and received antibiotics. No grade ≥ 3 complication occurred in the remaining patients during or immediately after the TAE and cryoablation procedure.

During the median follow-up period of 18 months (range 7–54 months), all patients were alive. The mean eGFR decreased gradually after combined cryoablation with TAE until one year (Table 3). Two (22%) of 9 patients (cases no.8 and no.9), whose pre-treatment eGFR was valued < 20 ml/min/1.73 m^2^ (19.8 and 12.8 ml/min/1.73 m^2^, respectively) before TAE, initiated hemodialysis at 3 and 19 months after treatment. In the other seven (78%) of 9 patients with ≥ 20 ml/min/1.73 m^2^ before TAE, the mean eGFR before treatments and at their last follow-up (median, nine months) were 26.8 ± 1.8 ml/min/1.73 m^2^ (range 24.1–29.2 ml/min/1.73 m^2^) and 20.6 ± 4.6 ml/min/1.73 m^2^ (range 10.0–23.6 ml/min/1.73 m^2^), respectively; the eGFR at their last follow-up was significantly decreased (p = 0.018). The eGFR decreased by a mean of 6.2 ± 5.3 ml/min/1.73 m^2^ (range 0.7–17.2 ml/min/1.73 m^2^), and the eGFR decrease rate was 22.5% ± 19.1% (range 2.9%–63.2%) at their last follow-up.

**Table 3:** Case details

Case	Age (y)	Tumor		Renal score	Contrast medium* (ml)	eGFR (ml/min/1.73 m^2^)						
		Size (cm)	Location			Pre-TAE	1 Day	3 Day	1 Mo	3 Mo	6 Mo	1 Year
1	61	28	Parenchymal	8	41	29.2	27.0	25.8	24.7	23.4	25.8	23.6
2	73	27	Parenchymal	9	128	28.4	–	31.1	33.9	36.1	31.6	26.4
3	53	19	Parenchymal	5	40	28.2	23.1	–	21.1	–	22.3	–
4	62	32	Mixed	8	88	27.2	25.0	23.3	16.4	–	10.0	–
5	64	17	Parenchymal	8	56	25.6	25.2	–	21.4	23.2	19.1	19.8
6	88	43	Mixed	9	45	24.9	22.8	–	22.7	24.2	23.6	–
7	64	32	Parenchymal	9	41	24.1	24.8	23.2	23.4	–	21.9	–
8†	52	23	Parenchymal	7	41	19.8	14.7	12.2	8.4	–	–	–
9‡	81	47	Mixed	10	40	12.8	–	10.4	10.3	8.5	7.7	7.4

^*^Amount of contrast medium used during transcatheter arterial embolization (TAE)

^†^Patient initiated on hemodialysis 3 months after treatment

^‡^Patient initiated on hemodialysis 19 months after treatment

*RENAL score* R.E.N.A.L. nephrometry score, *eGFR* estimated glomerular filtration rate

The imaging modalities periodically used for evaluating local tumor progression were MRI and CT in five patients, MRI alone in three (Fig. 1d), and CT alone in one. Except for one patient followed up by CT alone, contrast enhancement was not used in these examinations. No local tumor progression occurred in any patient.

## Discussion

Severe CKD patients, such as those with stage 4 or 5 CKD, are known to be at risk for various diseases, regardless of whether they have RCC. A previous systematic review reported that the risk of all-cause and cardiovascular death is increased in non-dialytic high-stage CKD patients [22]. The risk of death, cardiovascular events, or hospitalization was 3.2-, 2.8-, or 2.1-times higher in patients with CKD stage 4, respectively, compared to those with normal renal function (eGFR ≥ 60 ml/min/1.73 m^2^) [23]. It has been reported that the eGFR of stage 4 or 5 non-dialysis CKD patients has a mean natural course decline of 2.3–2.65 ml/min/1.73 m^2^ per year [24, 25]. Cohort studies reported that patients with stage 4 or higher CKD had a 24%–40% chance of initiation of dialysis within the first 2 years, and for stage 4 or 5 non-dialytic CKD patients with RCC, an even earlier induction of dialysis may be required.

Patients with T1a RCC are usually ideal candidates for surgery, especially for partial nephrectomy. Although less-invasive laparoscopic or robotic-assisted surgery has been recently used for partial nephrectomy, treatment of T1a RCC in severe CKD patients remains challenging in renal function preservation. Some authors reported that the eGFR decrease rate after partial nephrectomy in various stages of CKD patients was 2.4%–11.3% in the short term [26, 27], and 6.2%–12.5% by the last follow-up [15, 28]. In fact, many operators usually hesitate to perform surgery for non-dialysis patients with severe CKD because of the risk of introducing dialysis after resection. However, the recent increase in less-invasive percutaneous ablation therapy, such as cryoablation and radiofrequency ablation, may have a great impact on treatment strategies for such patients. Ablation therapy can be performed with conscious sedation under local anesthesia, making it suitable for patients who are unable to undergo general anesthesia [6, 13]. Procedural complications are usually asymptomatic and minor. Additionally, Chan et al. reported that in eGFR after treatment, T1 RCC patients undergoing ablation therapy (n = 103) had a significantly smaller decrease than those undergoing laparoscopic partial nephrectomy (n = 93) [29].

There are limited reports examining cryoablation of RCC in patients with severe CKD. One multicenter study evaluated renal functional outcomes after percutaneous cryoablation in patients with stage 4 or 5 CKD [14]. In 17 non-dialysis CKD patients, the eGFR decrease at three months postoperative was 2.2 ± 2.6 ml/min/1.73 m^2^ (decrease rate 10.6% ± 11.9%). Local tumor progression occurred in 2 (12%) of 17 patients in their cohort. They speculated that the ablation margin might have been insufficient in these two patients. In our cohort, two patients whose pre-treatment eGFR was < 20 ml/min/1.73 m^2^ required hemodialysis initiation, and there was an eGFR decrease of 6.4 ± 5.1 ml/min/1.73 m^2^ (decrease rate 22.5% ± 19.1%) in the other seven patients. Although our eGFR decrease was greater than that of cryoablation alone in a multicenter study [14], no local tumor progression was observed in this study. Prior TAE using iodized oil allowed accurate tumor localization, possibly contributing to securing appropriate ablation margins under CT fluoroscopy guidance.

As an adjunctive therapy for RCC before cryoablation, TAE can enhance tumor localization during ablation, improve local tumor control, and reduce renal hemorrhage [6–9, 28]. Michimoto et al. described TAE before cryoablation in 17 patients with small (size range, 12–36 mm) and endophytic RCCs [8]. They performed TAE one to five days before cryoablation with a mixture of ethanol and iodized oil. TAE was technically successful in 16 of 17 patients (one patient did not have an identifiable tumor-feeding artery on angiography.), and cryoablation was technically successful in all patients. The local control rate of RCC was 93% at a mean follow-up of 15.4 months. Gunn et al. retrospectively examined combined TAE with cryoablation in a group of nine patients, and a cryoablation-only group of 18 patients using propensity score matching analysis. Combined TAE with cryoablation did not increase complications or unfavorable effects on patients’ eGFR [30]. We have studied patients with stage 4 or 5 CKD, and although the eGFR at their last follow-up was significantly decreased as compared to that before combined cryoablation with TAE, the eGFR decrease was considered acceptable.

Although the use of iodized contrast medium for CKD patients is generally contraindicated because of the risk of contrast-induced nephropathy [13], the merits of TAE prior to cryoablation as described above are also known. Considering the risks and benefits, TAE using a contrast medium is performed after giving sufficient informed consent to the patient. In this study, six out of nine cases were RCC of the parenchymal type, and it is difficult to identify the extent of the tumor during ablation without TAE prior to cryoablation. Although it is possible to obtain localization of target lesions using intravenous contrast medium immediately before and during cryoablation, higher contrast medium use is expected compared to TAE prior to cryoablation. Although several studies have reported that relatively lower doses (< 125 mL) of contrast tend to be safer, they are not free of risk [31–34]. In this study, the mean amount of contrast medium used during TAE was 58 ± 29 ml (range 40–128 ml), which was the minimum required. The value of CO_2_ angiography in renal vascular disease is well established and may be a preventive measure against the development of contrast-induced nephropathy in patients with severe CKD [35]. Although CO_2_ cannot be used to deliver a liquid embolic agent, the use of CO_2_ angiography to advance the catheter to the tumor-feeding artery may enable embolization with a smaller contrast medium.

This retrospective study had some limitations. First, not all tumors were histologically diagnosed. Second, multiple types of embolic material were used. Third, we investigated a small number of patients for a short follow-up period. Forth, the imaging modalities and time period for follow-up were not uniform. Last, since we did not compare cryoablation alone and cryoablation combined with prior TAE, we could not fully evaluate the necessity of TAE.

In conclusion, percutaneous cryoablation combined with prior TAE for RCC in selected non-dialysis patients with stage 4 or 5 CKD was an effective therapy, with an acceptable impact on renal function in our small study cohort. Nevertheless, careful follow-up of renal function is mandatory, especially in patients with an eGFR value of < 20 ml/min/1.73 m^2^.
